# Effects of a priming session with heavy sled pushes on neuromuscular performance and perceived recovery in soccer players: a crossover design study during competitive microcycles

**DOI:** 10.5114/biolsport.2025.139082

**Published:** 2024-05-24

**Authors:** Víctor Pino-Mulero, Marcos A Soriano, Francisco Giuliano, Jaime González-García

**Affiliations:** 1 Strength Training and Neuromuscular (STreNgthP) Research Group. Camilo José Cela University. Faculty of Health Science Madrid, Spain; 2 Centre for Sport Studies, Rey Juan Carlos University, 28943 Madrid, Spain; 3 Centre for Human Movement and Rehabilitation, University of Salford, Frederick Road Campus, Manchester, UK; 4 Exercise and Sport Sciences, Faculty of Health Science, Universidad Francisco de Vitoria, 28223 Pozuelo, Spain

**Keywords:** Priming, Performance, Football, Soccer, Sprint, Sled, Push, Recovery, Traning load

## Abstract

This study compared the effects of a sled push priming session on performance in 20 m sprint times, vertical jump, and perceived recovery, in comparison to a control condition, during competitive microcycles. Sixteen young semi-professional football players completed two conditions: priming (heavy sled pushes and MD-1 training) and control (only MD-1 regular training), in a crossover design. Twenty-metre sprint times and countermovement jump (CMJ) were assessed 24 hours following the completion of the experimental sessions. The distribution of training load (TL) was similar between the two microcycles on each training day (p > 0.506). At 24 hours, 20 m sprint times were lower (p < 0.001; ES = -1.41), and jump height increased (p = 0.001; ES = 1.11) in the priming condition compared to the control. Individual response analysis showed that 62.5% of the participants ran faster, and 81.3% jumped higher 24 h after priming compared to the control. Furthermore, the added sled push priming exercise did not alter the recovery values perceived by players at 24 hours (p = 0.310). These findings support the use of priming exercises within competitive microcycles by incorporating these sessions on MD-1, as they appear to improve 20 m sprint time and vertical jump without impacting perceived recovery. These results contribute to the understanding of the effects of a low-volume priming session based on heavy sled push on delayed potentiation in sprint and CMJ.

## INTRODUCTION

Soccer is a team sport with a high technical and tactical component, non-cyclical and intermittent in nature. It involves the interweaving of activities of maximum short-duration intensity, such as 10–20 m sprint runs, with high-intensity actions, along with periods of lower and moderate-intensity activities [1]. Specifically, modern soccer has evolved in recent years in terms of game speed, evident in the increase in ball speed, player density (i.e. congestion of players around the ball) and high-intensity and sprint distances [2]. These high-intensity activities, especially straight sprinting and jumping [3], occur during decisive moments in the game, potentially influencing the final score [3]. Therefore, coaches and researchers should seek methods that optimize the sprinting and jumping capacities of soccer players during competition.

Resisted sprints are an effective training method for improving sprint performance in various populations in the long term [4]. This type of training requires high production of horizontal force to over-come the resistance of the sled’s mass [5]. Sled pushing possesses unique characteristics that differentiate it from sled pulling [5], yet research on sled pushing is scarce [6]. The actions of pulling and pushing sleds, respectively, primarily differ in how they provide a posterior and anterior loading stimulus on the athlete, as well as the technical differences [5]. During sled pushing, there is a more anteriorized position, and the arms are used to push the sled forward during the drive phase, in addition to experiencing higher friction compared to sled pull due to its larger contact surface. Finally, the more forward position during sled pushing may influence the activation of certain muscle groups and sprint mechanics [5], although it has not been directly analysed. This can impact the sprint resistance profile and the velocity decrement [5], as well as presenting different potentiation effects and acute recovery profile after training. Despite technical differences with sled pushing, recovery kinetics of resisted sled pulls with low loads (20%BM) have shown performance impairments, coupled with exercise-induced muscle damage (EIMD) and reductions in peak eccentric force of knee flexors and extensors, 24 hours after a training volume of 210 m [7]. In contrast, when the training volume is reduced (160 m), irrespective of external load (0%BM to 80%BM), both jump height and 10 and 20 m sprint times exhibit complete recovery after 24 hours of rest. These results suggest that low volumes of resisted sled pull provide sufficient recovery at 24 hours, ensuring that sprinting is not limited by the preceding training session. However, to the best of our knowledge, there is no study describing the recovery profile following sled push training.

While it has been demonstrated that the implementation of longterm resistance and resisted sprint training enhances jumping, acceleration and sprinting [8, 9], there also exists an opportunity to acutely enhance performance through conditioning activities (CA) [10, 11], especially in athletes with higher levels of muscular strength [10, 12, 13]. This opportunity can be important in contexts where dedicating extensive hours to strength training is not feasible, especially within high-level sport contexts. In contrast to other preconditioning training methods such as post-activation performance enhancement (PAPE) [14], ‘priming’ exercises are conditioning activities commonly per formed on the same day [10, 11, 15–19] or the day prior [12, 17, 18, 20, 21] to a competition/task, aiming to induce enhancing performance effects over the subsequent 2–48 hours [22]. According to Harrison et al., [23] 51% of training professionals in various sports have confirmed the application of priming exercises, even though the beneficial training configurations (type of exercise, intensity, volume, level of effort, etc.) and recovery windows have not been clearly identified [11, 15, 19, 21, 24]. Previous studies have examined the priming effect after diverse set configurations and recovery durations resulting in inconclusive outcomes [13, 15, 18, 21, 24] and suggesting interindividual variability on recovery and delayed potentiation enhancement in team sport in males and females [10–12, 19]. Hence, additional research is essential to investigate potentially beneficial priming exercise configurations for enhancing sports performance.

To achieve a delayed performance enhancement effect, a positive net balance between potentiation and fatigue and recovery is necessary [22]. In this regard, Seitz et al. [25] identified that a single sled push with 75% of body mass over 15 m significantly improved sprint times from 8 minutes onward (ES = -0.36 to -0.42) after conditioning activity (CA). Meanwhile, sled pushing with 125% of body mass over 9 m showed increases in 20 m sprint time immediately after (ES = 0.64) and up to 12 minutes after CA (ES = 0.34).

Sled pushes with heavy loads, although not excessively heavy (< 125% of body mass), have shown an acute improvement in 20 m sprint performance [25]. Additionally, this type of training stimulus has demonstrated a recovery window of approximately 24 hours [26]. Therefore, these findings suggest that a low-volume priming session focused on heavy sled pushes could be an effective strategy to achieve performance improvements after 24 hours of recovery. Furthermore, all previous research shares a common limitation that is addressed in the current study. None of them have controlled the training load imposed on athletes throughout the training microcycles. Therefore, the objectives of the present investigation are: i) to identify the effects of a priming session performed 24 hours before competition based on a heavy resisted sled push on 20 m sprint and vertical jump performance, and ii) to compare the microcycle training loads and perceived recoveries between the priming and control conditions during experimental weeks. We anticipated that the priming session could improve 20 m sprint times. Moreover, considering the different responses previously observed in vertical jump performance, we hypothesized that there would be a different response among participants. Additionally, given the low-volume nature of the priming exercises, we hypothesized that this session would not have an impact on the participants’ perceived recovery.

## MATERIALS AND METHODS

A randomized and counterbalanced repeated measures approach was employed to investigate the possible delayed improvement in performance resulting from the priming exercise. Both the 20 m sprint performance and the countermovement jump (CMJ) height and jump momentum were assessed at three different time points for all the soccer players, in addition to familiarization testing. The first day served as a baseline session. It was conducted one week prior to session 2. Sessions 2 and 3 were used for the implementation of either the priming or control condition, depending on each athlete’s assignment. Experimental conditions were separated by 1 week. For all conditions, participants were allowed breakfast but were asked to refrain from alcohol and caffeine for 24 hours prior to the session. In both experimental conditions, athletes were instructed to maintain the same nutritional intake and to abstain from consuming alcohol or caffeine in the 24 hours leading up to the assessments.

### Participants

An a-priori sample size estimation revealed that a minimum of 4 participants were required for a within-factors repeated measures ANOVA assuming a partial eta-squared (*η*^2^) of 0.66 for sprint time after sled PAPE, with a repeated measures Pearson’s correlation of 0.86 and values of 5% and 1% for type I and type II errors, respectively. However, sixteen young soccer players belonging to the top U-18 category in Spain were recruited for this study to conduct an analysis of individual responses with an adequate number of participants (mean ± SD: body mass (BM): 68.3 ± 6.0 kg; height: 1.80 ± 0.10 m; age: 18 ± 1 years; resistance training experience: 3 ± 1 years). The study and the informed consent procedures were reviewed and approved by the Research Ethics Committee (16_23_ RNM_FP), in compliance with the most recent version of the Declaration of Helsinki. Prior to signing the institutionally approved informed consent document to participate in the study, subjects were fully informed about the investigation’s advantages and potential risks.

### Experimental design

**FIG. 1:**
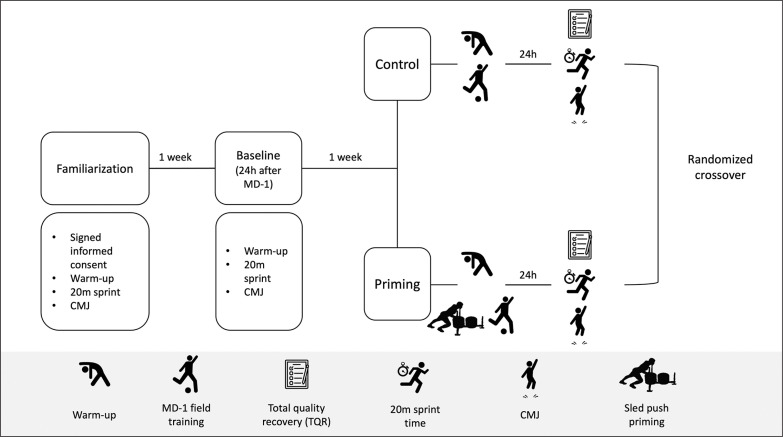
Flow chart of the study.

Figure 1 illustrates the investigation’s flowchart. Participants were assessed on four occasions. The first session served as familiarization, the second as a baseline, and finally there were two experimental sessions—one at 24 hours after the control condition and one at 24 hours after priming. In each evaluation session, two 20 m sprints were performed, followed by three CMJs. Three minutes of recovery were provided both between repetitions and between assessments.

During familiarization, participants signed the informed consent and performed a standardized warm-up. This warm-up consisted of 3 minutes of global joint mobility exercises, followed by 3 minutes of dynamic stretching (frontal and lateral swings, 10 repetitions on each leg). Additionally, participants performed 3 sets of 3 repetitions of bodyweight exercises (horizontal jumps). Finally, two sets of two 20 m sprints with 20% body mass (BM) were completed before warm-up ended. This warm-up remained the same during all testing sessions.

The inter-day reliability was calculated using the data obtained from the baseline session and the control condition. Two randomized conditions were implemented throughout the investigation: 1) control, where participants conducted a traditional MD-1 field training session, and 2) priming, where participants engaged in a complementary sled push session to the MD-1 field training. In both conditions and following a 24-hour recovery period, the CMJ unloaded 20 m sprint times, and perceived recovery quality were assessed. To avoid any effect of circadian rhythms on recovery and performance, participants were scheduled at the same time in both experimental conditions [27].

### Sled push priming exercise

After the warm-up, in the priming condition, each participant performed 2 sets of 2 repetitions with heavy sled pushes with 100% of their body mass over 15 m, with 30 seconds between repetitions and 3 minutes of recovery between sets. Mechanical work was calculated as previously (mechanical work = load × distance) [25], giving that the sled push was conducted on an artificial turf surface. The starting position of the soccer players was standardized following the instructions of Seitz et al. [25]. The sled weighed 40 kg without any added load (Evergy Sled, Madrid, Spain). Calibrated bumper plates (Singular WOD, Madrid, Spain) were added to reach a total load equal to 100% of the participant’s body weight. These bumper plates ranged from 1 kg to 20 kg in weight. Moreover, for the sled’s 15 m resisted displacement to be considered valid, it was required to fully traverse the designated lines on the ground. This criterion received particular attention during the familiarization process, which also included guidance on hand placement and the directive to exert maximum effort. While the exercise protocol was carried out in the priming condition, in the control condition, the players engaged in several rondos to introduce the training session. This is a training exercise in nearly static positions, where a group of players forms a circle to pass the ball among themselves, developing technical skills and the ability to maintain ball possession. It is commonly used in football to introduce players to the main session.

### 20 m sprint times and CMJ

Twenty-four hours after the completion of the priming session and MD-1 field training (or only MD-1 field training in the control condition), the sprint performance in a 20 m sprint and CMJ vertical height were assessed. The evaluation of 20 m sprint times was conducted using electronic timing gates (Witty-gate photocells, Bolzano, Italy) positioned at 0 and at 20 m. Two sprints were performed with a 3-minute rest between attempts. All players initiated the sprint from a semi-crouched position with their front foot 50 cm away from the starting line [28]. The timer started when the soccer player broke the first infrared beam of the starting line [25]. Players were verbally encouraged to run as fast as possible.

To identify the possible delayed effects produced by the priming exercise on jump metrics, each participant performed three CMJs using a Hawkin Dynamics Inc. (Westbrook, Maine, USA) dual Force-Platform at a sampling rate of 1,000 Hz. The calculation of centre-of-mass (COM) velocity involved dividing the vertical force (adjusted for body weight) by body mass and subsequently integrating the product using the trapezoid rule. COM displacement was ascertained through double integration of the vertical force data [29]. Jump momentum was calculated by multiplying the subject’s mass by the take-off velocity. The jump was deemed successful when executed with arms akimbo, and participants stayed still for at least one second during the weighing phase [30]. Jump height and jump momentum were selected for analysis.

### Training load and recovery

The measurement of internal player training load (TL) involved using session rating of perceived exertion (sRPE), which was calculated by multiplying the RPE value by the session’s duration in minutes. This calculation was applied to every training session, including the recovery periods between exercises and warm-up [31]. Players completed an online questionnaire consisting of a single question: “How hard was your physical exertion during the session?” They provided their responses using their personal mobile phones, and the data were stored using a cloud-based software platform (Google Forms, California, United States of America). The 10-point RPE Borg scale was administered 15–30 minutes after both training sessions and matches [32].

This method has demonstrated a stronger correlation with heart rate-based training impulse (TRIMP) when compared to traditional paper-and-pencil methods [33]. This suggests that sRPE serves as a valid indicator of the overall internal load in soccer [34].

**FIG. 2:**
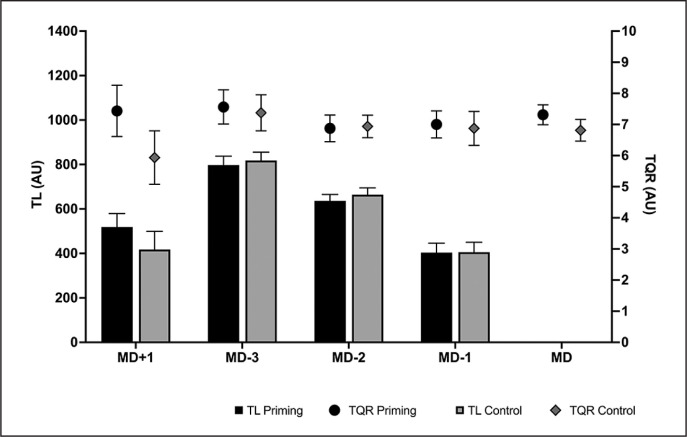
Training load (TL) and Total Quality Recovery Scale (TQR) throughout the two microcycles of the experimental conditions. TL = Training load; TQR = Total Quality Recovery; MD = Match day

It is worth noting that all players had prior familiarity with the RPE scale, having been acquainted with it for a minimum of two years. For a detailed depiction of the training load (TL) in both experimental microcycles, see Figure 2.

The players’ perceived recovery status was assessed using the Total Quality Recovery (TQR) scale [35]. Each player was asked to respond regarding their subjective perceived recovery on a scale ranging from 0 (indicating very, very poor recovery) to 10 (indicating very, very good recovery). Participants completed a custom-made questionnaire via Google Forms. They filled it out in the morning, 30 minutes after waking up, always between 7:00 AM and 9:00 AM. The data from this questionnaire were stored in a cloud-based Excel sheet, where it was confirmed that the data were collected according to the mentioned criteria. All participant responses met the inclusion criteria for analysis. Participants were familiar with the process as it was part of their daily training routine. The TQR values of the two experimental microcycles are also displayed in Figure 2.

**FIG. 3:**
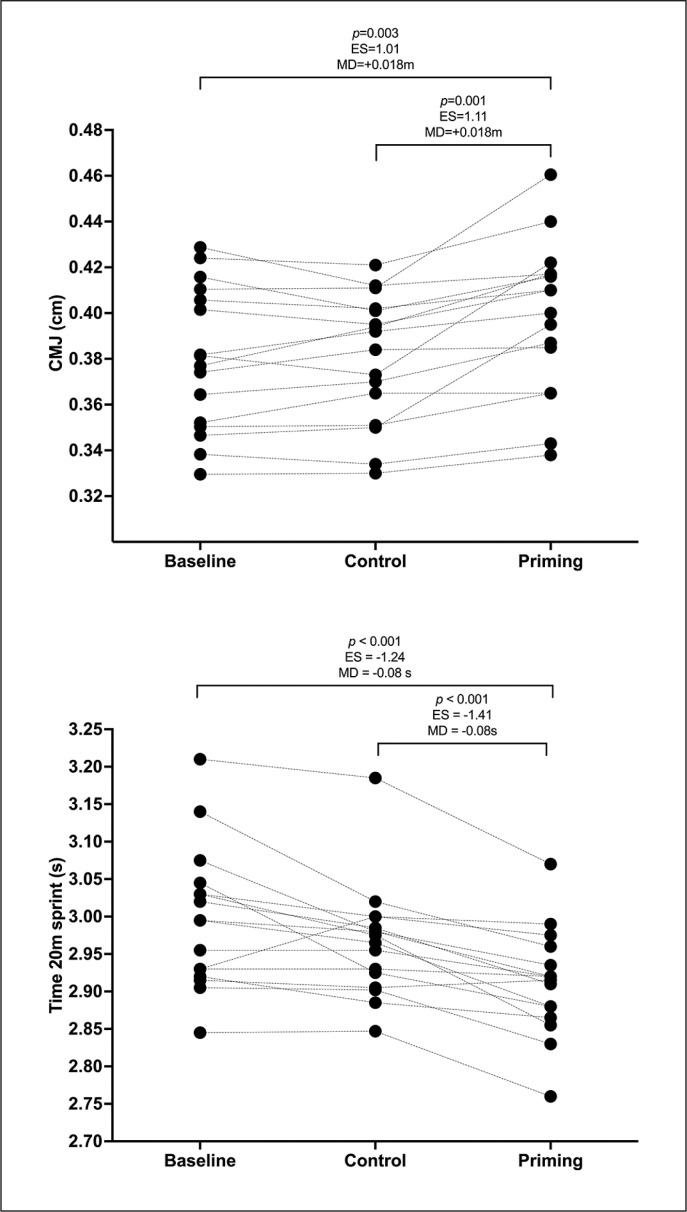
Individual differences in CMJ and 20m sprint times between experimental conditions. ES = Effect size; MD = Mean difference

### Statistical analysis

Statistical analyses were conducted using IBM SPSS Statistics version 26.0 (IBM Corp., Armonk, NY, EE. UU.). Data collected during the familiarization phase exhibited a normal distribution. If necessary, Greenhouse-Geisser sphericity correction was used. The evaluation of intraclass correlation coefficients (ICC) with 95% confidence intervals (95% CI) followed these criteria: poor reliability (< 0.5), moderate reliability (0.5–0.75), good reliability (0.75–0.90), and excellent reliability (> 0.90) [36]. Additionally, the standard error of measurement (SEM) and coefficient of variation (CV%) were calculated to identify absolute reliability. Participants who exhibited positive, negative, or non-responsive reactions to the priming exercise were identified based on whether the absolute change fell below or exceeded the smallest worthwhile change (SWC = 0.2 × between-subject SD) [21, 37]. One-way repeated measures analysis of variance (ANOVA), with 95% confidence intervals (95% CI), was conducted to assess the impact of the priming intervention in comparison to control and familiarization data. Two-way repeated measures ANOVA was also conducted to identify potential differences in TL and TQR during the microcycles of the experimental protocols. Pairwise comparisons were examined using Bonferroni’s post-hoc test. Furthermore, effect sizes (Cohen’s d) were computed between conditions and categorized as follows: ≤ 0.2 (trivial), ≥ 0.2–0.6 (small), ≥ 0.6–1.2 (moderate), ≥ 1.2–2.0 (large), and ≥ 2 (very large) [38]. The results are presented as mean ± standard deviation (SD), and the significance level was set at p < 0.05.

## RESULTS

20 m sprint time showed good reliability (ICC [95%CI] = 0.86 [0.64 to 0.95]; SEM = 0.04 s; CV = 2.73%). Similarly, jump height (ICC [95%CI] = 0.96 [0.88 to 0.98]; SEM = 0.007 m; CV = 7.76%), and jump momentum (ICC [95%CI] = 0.98 [0.93 to 0.99]; SEM = 2.38 kg*m/s; CV = 7.78%) showed excellent reliability.

Main effects of the microcycle session day (MD) were observed in both TQR (F_4,60_ = 2.97; p = 0.027; $\eta_{\text{p}}^{2}$ = 0.165) and TL 2 (F_3,45_ = 139.19; p < 0.001; *η*_p_ = 0.903). Additionally, for TQR, there was also a main effect of condition (F_1,60_ = 7.28; p = 0.017; 2 *η*_p_ = 0.327) and an MD × condition interaction effect (F_4,60_ = 2.81; 2 p = 0.033; *η*_p_ = 0.158). The comparison of training loads between the microcycles of both conditions revealed that there were no differences on any of the training days (p > 0.506). Furthermore, the evolution of training load over the days followed the same pattern in both conditions. The highest load values were observed on MD-3, with higher sRPE than on days MD+1 (priming: p < 0.001; control: p < 0.001), MD-2 (priming: p < 0.001; control: p < 0.001), and MD-1 (priming: p < 0.001; control: p < 0.001). In both groups, MD-2 showed a higher training load than MD-1 priming: (p < 0.001; control: p < 0.001). Similar to TL, no differences were observed in TQR between any of the conditions on any of the days assessed (p > 0.310). In both conditions, the day with higher perceived recovery was MD-3 (Figure 2).

A main effect of condition was observed in both jump height 2 (F_2,30_ = 15.3; p < 0.001; *η*_p_ 2 = 0.506) and 20 m sprint times (F_2,30 =_ 20.9; p < 0.001; *η*_p_ = 0.582) but not in jump momentum (p > 0.05). The differences observed in the experimental conditions are displayed in Figure 3. Both the 20 m sprint times and jump height indicate better performance at 24 h after the priming condition compared to the control and baseline conditions, with no differences between the latter two groups (p > 0.05).

## DISCUSSION

This study aimed to investigate the effects of a heavy sled push priming session on 20 m sprint and CMJ performance. Additionally, we aimed to assess and compare the influence of this session on perceived recovery during the competitive microcycles of the experimental period. The results obtained in this research are in line with the hypothesized outcomes. The implementation of a priming session involving low-volume heavy sled pushes performed 24 hours before competition led to significant improvements in 20 m sprint times and jump height, as anticipated in our hypothesis. Furthermore, the TQR values indicate that this priming exercise configuration does not affect the perception of recovery after 24 hours of rest during microcycles with similar TL distribution during their normal field training.

This is the first study that has examined the effects of a heavy sled push priming session on sprint and CMJ performance, making it challenging to compare the results. To discuss our findings, we have referred to studies that examined the PAPE effects after heavy sled push [25], studies about the recovery profile after different sled pulls loads (even if they were not priming sessions) [26, 39] and studies on priming but with either unresisted sprints or sprints resisted with light loads [24, 40]. Monahan et al. [39] analysed the physiological and perceptual response in athletes from various team sports to a session of resisted sprints with loads of approximately ~12.6% and 33.7% of body mass. After 12 repetitions of 20 m (240 m total), they observed that the protocol with a higher load (i.e., 33.7% of body mass) was more demanding in terms of internal load (higher heart rate, greater lactate concentrations and higher RPE) than the session with a lower %BM. Similarly, Bachero-Mena et al. [26] also observed higher fatigue indexes and internal responses to training as the external resistance applied to the sled pull training increases (from 0% BM to 80% BM). In this case, the heaviest load (80% BM) showed the highest fatigue index (6.1 ± 2.1%), the highest lactate concentrations after 4 and 8 minutes, as well as reductions in CMJ height and 20 m sprint performance. Although these results may suggest that the use of very heavy loads may not be an effective method to enhance neuromuscular performance (as they present the highest levels of fatigue) [22, 41], strength and conditioning professionals need to consider the recovery window until the next training and/or competition and the players’ characteristics [41]. In these terms, both studies demonstrated that the analysed mechanical performance markers (20 m sprint time and CMJ) of team sport players [39] and physical active individuals [26] returned to baseline levels after 24 hours of recovery, despite using higher training volumes than in the present research (160 and 240 m vs. 60 m). Therefore, using lower training volumes could ensure complete recovery, allowing for an increased net balance between potentiation and fatigue to yield benefits in sports performance [22]. Indeed, TQR data confirm that participants in this study reported complete subjective recovery after the priming exercise (Figure 2).

Regarding delayed potentiation effects, our findings revealed a reduction in the 20 m sprint time by 1.8% ± 1.3% and an increase in CMJ jump height by 3.9 ± 3.1%, but not jump momentum, compared to the control condition. Despite the discrepancy in recovery durations (8–12 minutes versus 24 hours) and the absence of a baseline immediately preceding the priming exercise in our study, the performance enhancements in the 20 m sprint times align with those observed after a post-activation potentiation (PAPE) protocol involving a resisted sled push covering 20 m with a load equivalent to 75%BM. This suggests that the application of a heavy, low-volume sled push protocol (2 sets × 2 repetitions × 15 m = 60 m) might yield benefits for sprint time comparable to those observed after a heavy sled push PAPE study [25]. Despite its potential to potentiate performance, not all sprint-based priming exercises have demonstrated a delayed potentiation effect on sprint times and/or CMJ metrics [17, 24, 40, 42]. The predominant body of research on sprint-based priming has been conducted during the morning [17, 24, 42], analysing the effects of delayed potentiation at 5–6 hours. According to these studies, the results of applying a non-resisted sprint-based priming exercise with changes of direction have shown a consistent increase in sprint performance after 5–6 hours of recovery [17, 24, 42].

Although the literature has predominantly analysed the effects of a priming exercise in the morning on afternoon performance, this temporal setup cannot be implemented if the competition occurs in the morning. To the best of our knowledge, just one study has observed the effects of a sled-based priming session after more than 6 hours of recovery on sprint performance [40]. They found that resisted sled pulls with a load of 10% of body weight moderately increased the 20 m time (ES = 0.59), observing 50 m times like baseline values due to an improvement in the 30 m flying sprint times but not in the 0–20 m split. These results from Kotula et al. [40] suggest that their resisted sled pull protocol is not effective for en-hancing performance in the initial 0–20 m phase during a sprint test. But it was for enhancing the high-speed sprint phase (20–50 m), which was also potentiated after an overspeed priming exercise, suggesting that potentiation after priming is movement- and velocity-specific [11, 20, 24, 42]. In this regard, the heavy sled push used as a priming exercise in this study has kinetic and kinematic characteristics that make it more similar to a march exercise rather than sprint [43], and this may lead to different potentiation effects that may not enhance performance in the high-speed phase but may have adequate transfer to the 0–20 m phase. In fact, sled pushes with very heavy resistance are performed at significantly slower speeds and under different muscular contraction conditions compared to conventional sprints. This allows for the generation of greater impulses in each step, acting as a training stimulus for maximal force production rather than force generation at high speeds [43]. Hence, a possible explanation for the observed potentiation phenomenon could be that very heavy resisted sprints may enhance sprint times due to an increase in horizontal impulse or thanks to a better technical capacity to orient the generated impulses in a more horizontal direction [44], as both mechanical capabilities are related to sprint and acceleration performance [45]. Moreover, potentiation following a heavy sled push may be attributed to an increase in neural activity. Although not directly evaluated, this mechanism has been proposed and could be involved in delayed potentiation [22], as activities with longer contraction durations (i.e., longer contact times due to high load) are associated with higher neural activity [46], which, in turn, may lead to increases in muscle activity.

It is necessary to consider the individual characteristics of the participants, as they play a mediating role in the response to exercise, affecting the recovery periods and delayed potentiation effects after priming. In fact, potentiation responses showed high interindividual variation. Our individual analysis revealed that 81.3% (13/16) of our participants improved their performance in the CMJ jump while 62.5% (10/16) improved sprint times above the SEM. This suggests that a low-volume, heavy sled push may serve as an appropriate training stimulus to induce a delayed potentiation effect on sprint times and CMJ height depending on the participant.

None of the previous priming studies have considered the training loads experienced by athletes during their field training. They have merely indicated the hours of recovery since the last training session. Therefore, one of the strengths of this study is the inclusion of the resisted sled push stimulus during a competitive microcycle in a “real-world” application context, considering the sRPE TL experienced by the athletes during their typical field training. This allows us to ensure that the TL endured by the participants in their field sessions, in addition to the priming session of the experimental protocol, has been similar in the priming and control conditions. However, this study has several limitations that need to be addressed in future research. One of the main limitations of the study is the absence of a baseline assessed 24 hours before the post-priming evaluation. This prevents understanding the participants’ vertical jump and 20 m sprint performance prior to the conditional activity, there-by limiting the analysis over time and, therefore, the validity of the results obtained 24 hours after priming. Additionally, the prescription of external load was based on the %BM method. This method can result in significant inter-subject variability in the velocity decrement relative to maximum sprint. This methodological issue may introduce uncertainties in the interpretation of the outcomes given the possible different training stimulus provided to each of the players. The sample consisted of 16 young soccer players; these results should be interpreted with caution if we attempt to extrapolate them to other populations. Furthermore, delayed recovery and/or potentiation were only assessed using physical performance variables or subjective recovery measures, without including any evaluation of objective recovery state (e.g., serum creatine kinase, capillary ammonia, voluntary activation, or muscle twitch response), thus limiting the understanding of the aetiology of fatigue and recovery profile of the participants. Indeed, we suggest that future research should analyse the possible mechanisms involved in these delayed performance improvements to generate more rigorous training protocols.

## CONCLUSIONS

In summary, our results confirm the effectiveness of a low-volume, heavy resisted sled push as a priming method during competitive microcycles in soccer, providing valuable insights into their influence on physical performance and athletes’ recovery perceptions, particularly in team sports contexts. These findings contribute to the growing body of knowledge regarding the strategic use of priming techniques to optimize athletic performance and to accumulate sprint training load during the microcycle without affecting recovery perception. Finally, we recommend monitoring acute responses outside the competitive period to identify positive responders, thereby minimizing potential negative effects on performance that may arise from the application of this heavy, low-volume sled push priming.

## References

[1] Di Salvo V, Baron R, Tschan H, et al. Performance characteristics according to playing position in elite soccer. Int J Sports Med. 2007; 28:222–227. doi: 10.1055/S-2006-924294. 17024626

[2] Allen T, Taberner M, Zhilkin M, et al. Running more than before? The evolution of running load demands in the English Premier League. Int J Sports Sci Coach 2024; 19(2): doi: 10.1177/17479541231164507.

[3] Faude O, Koch T, Meyer T. Straight sprinting is the most frequent action in goal situations in professional football. J Sports Sci. 2012; 30:625–631. doi: 10.1080/02640414.2012.665940. 22394328

[4] Alcaraz PE, Carlos-Vivas J, Oponjuru BO, et al. The Effectiveness of Resisted Sled Training (RST) for Sprint Performance: A Systematic Review and Meta-analysis. Sports Med. 2018; 48:2143–2165. doi: 10.1007/S40279-018-0947-8. 29926369

[5] Cahill MJ, Cronin JB, Oliver JL, et al. Sled Pushing and Pulling to Enhance Speed Capability. Strength Cond J. 2019; 41:94–104. doi: 10.1519/SSC.0000000000000460.

[6] Cahill MJ, Oliver JL, Cronin JB, et al. Influence of resisted sled-push training on the sprint force-velocity profile of male high school athletes. Scand J Med Sci Sports. 2020; 30:442–449. doi: 10.1111/SMS.13600. 31742795

[7] Liakou CA, Fatouros IG, Poulios A, et al. Recovery kinetics following sprint training: resisted versus unresisted sprints. Eur J Appl Physiol. 2023; 1–16. doi: 10.1007/S00421-023-05317-X/TABLES/5. PMC10879260 37776346

[8] Cormie P, McBride JM, McCaulley GO. Power-Time, Force-Time, and Velocity-Time Curve Analysis of the Countermovement Jump: Impact of Training. J Strength Cond Res. 2009; 23:177–186. doi: 10.1519/JSC.0b013e3181889324. 19077740

[9] Lesinski M, Prieske O, Granacher U. Effects and dose-response relationships of resistance training on physical performance in youth athletes: A systematic review and meta-analysis. Br J Sports Med. 2016; 50:781–795. 26851290 10.1136/bjsports-2015-095497 PMC4941165

[10] González-García J, Latella C, Aguilar-Navarro M, et al. Effects of Resistance Priming Exercise on Within-day Jumping Performance and its Relationship with Strength Level. Int J Sports Med. 2023; 44:38–47. doi: 10.1055/A-1898-4888. 35820447

[11] González-García J, Giráldez-Costas V, Ruiz-Moreno C, et al. Delayed potentiation effects on neuromuscular performance after optimal load and high load resistance priming sessions using velocity loss. Eur J Sport Sci. 2020; 1–28. doi: 10.1080/17461391.2020.1845816. 33135577

[12] Nishioka T, Okada J. Influence of Strength Level on Performance Enhancement Using Resistance Priming. J Strength Cond Res. 2021; Publish Ah. doi: 10.1519/JSC.0000000000004169. PMC8677605 34711771

[13] Donghi F, Rampinini E, Bosio A, et al. Morning Priming Exercise Strategy to Enhance Afternoon Performance in Young Elite Soccer Players. Int J Sports Physiol Perform. 2021; 16:407–414. doi: 10.1123/IJSPP.2020-0094. 33401241

[14] Blazevich AJ, Babault N. Post-activation Potentiation Versus Post-activation Performance Enhancement in Humans: Historical Perspective, Underlying Mechanisms, and Current Issues. Front Physiol. 2019; 10:1359. doi: 10.3389/fphys.2019.01359. 31736781 PMC6838751

[15] Rud B, Øygard E, Dahl EB, et al. The Effect of Resistance Exercise Priming in the Morning on Afternoon Sprint Cross-Country Skiing Performance. Int J Sports Physiol Perform. 2021; 1–8. doi: 10.1123/ijspp.2020-0881. 34021095

[16] Dahl EB, Øygard E, Paulsen G, et al. Morning Preconditioning Exercise Does Not Increase Afternoon Performance in Competitive Runners. Int J Sports Physiol Perform. 2021; 1:1–8. doi: 10.1123/ijspp.2020-0747. 34044367

[17] Cook CJ, Kilduff LP, Crewther BT, et al. Morning based strength training improves afternoon physical performance in rugby union players. J Sci Med Sport. 2014; 17:317–321. doi: 10.1016/j.jsams.2013.04.016. 23707139

[18] Mason BRJ, Argus CK, Norcott B, et al. Resistance Training Priming Activity Improves Upper-Body Power Output in Rugby Players: Implications for Game Day Performance. J Strength Cond Res. 2017; 31:913–920. doi: 10.1519/JSC.0000000000001552. 27386962

[19] González-García J, Aguilar-Navarro M, Giráldez-Costas V, et al. Time Course of Jump Recovery and Performance After Velocity-Based Priming and Concurrent Caffeine Intake. Res Q Exerc Sport. 2022; Publish ah.: 1–13. doi: 10.1080/02701367.2022.2041162. 35442175

[20] Tsoukos A, Veligekas P, Brown LE, et al. Delayed effects of a low-volume, power-type resistance exercise session on explosive performance. J Strength Cond Res. 2018; 32:643–650. doi: 10.1519/jsc.0000000000001812. 28291764

[21] Harrison PW, James LP, Jenkins DG, et al. Time Course of Neuromuscular, Hormonal, and Perceptual Responses Following Moderate- and High-Load Resistance Priming Exercise. Int J Sports Physiol Perform. 2021; 1–11. doi: 10.1123/ijspp.2020-0646. 33761461

[22] Harrison PW, James LP, McGuigan MR, et al. Resistance Priming to Enhance Neuromuscular Performance in Sport: Evidence, Potential Mechanisms and Directions for Future Research. Sports Med. 2019; 49:1499–1514. doi: 10.1007/s40279-019-01136-3. 31203499

[23] Harrison PW, James LP, McGuigan MR, et al. Prevalence and application of priming exercise in high performance sport. J Sci Med Sport. 2019; doi: 10.1016/j.jsams.2019.09.010. 31594712

[24] Russell M, King A, Bracken RM, et al. A comparison of different modes of morning priming exercise on afternoon performance. Int J Sports Physiol Perform. 2016; 11:763–767. doi: 10.1123/ijspp.2015-0508. 26658460

[25] Seitz LB, Mina MA, Haff GG. A sled push stimulus potentiates subsequent 20-m sprint performance. J Sci Med Sport. 2017; 20:781–785. doi: 10.1016/J.JSAMS.2016.12.074. 28185808

[26] Bachero-Mena B, Sánchez-Moreno M, Pareja-Blanco F, et al. Acute and Short-Term Response to Different Loading Conditions During Resisted Sprint Training. Int J Sports Physiol Perform. 2020; 15:997–1004. doi: 10.1123/IJSPP.2019-0723. 32473591

[27] Pallarés JG, López-Samanes Á, Moreno J, et al. Circadian rhythm effects on neuromuscular and sprint swimming performance. Biol Rhythm Res. 2013; 45:51–60. doi: 10.1080/09291016.2013.797160.

[28] Winwood PW, Posthumus LR, Cronin JB, et al. The acute potentiating effects of heavy sled pulls on sprint performance. J Strength Cond Res. 2016; 30. doi: 10.1519/JSC.0000000000001227. 26439786

[29] Moir GL. Three different methods of calculating vertical jump height from force platform data in men and women. Meas Phys Educ Exerc Sci. 2008; 12:207–218. doi: 10.1080/10913670802349766.

[30] McMahon JJ, Suchomel TJ, Lake JP, et al. Understanding the key phases of the countermovement jump force-time curve. Strength Cond J. 2018; 40:96–106. doi: 10.1519/SSC.0000000000000375.

[31] Alexiou H, Coutts AJ. A comparison of methods used for quantifying internal training load in women soccer players. Int J Sports Physiol Perform. 2008; 3. doi: 10.1123/ijspp.3.3.320. 19211944

[32] Foster C, Florhaug JA, Franklin J, et al. A New Approach to Monitoring Exercise Training. J Strength Cond Res. 2001; 15. doi: 10.1519/1533-4287(2001)015<0109:ANATME>2.0.CO;2. 11708692

[33] Roos L, Taube W, Tuch C, et al. Factors that influence the rating of perceived exertion after endurance training. Int J Sports Physiol Perform. 2018; 13. doi: 10.1123/ijspp.2017-0707. 29543071

[34] Impellizzeri FM, Rampinini E, Coutts AJ, et al. Use of RPE-based training load in soccer. Med Sci Sports Exerc. 2004; 36. doi: 10.1249/01.MSS.0000128199.23901.2F. 15179175

[35] Kenttä G, Hassmén P. Overtraining and recovery. A conceptual model. Sports Med 1998; 26:1–16. doi: 10.2165/00007256-199826010-00001. 9739537

[36] Portney LG, Watkins MP. Foundations of Clinical Research: Applications to Practice, 3rd Edition | Pearson. New Jersey; 2008.

[37] Hopkins WG. How to Interpret Changes in an Athletic Performance Test. Sportscience. 2004; 8.

[38] Hopkins WG, Marshall SW, Batterham AM, et al. Progressive statistics for studies in sports medicine and exercise science. Med Sci Sports Exerc. 2009; 41:3–12. doi: 10.1249/MSS.0b013e31818cb278. 19092709

[39] Monahan M, Petrakos G, Egan B. Physiological and Perceptual Responses to a Single Session of Resisted Sled Sprint Training at Light or Heavy Sled Loads. J Strength Cond Res. 2022; 36:2733–2740. doi: 10.1519/JSC.0000000000003973. 36135030

[40] Kotuła K, Matusiński A, Zając A, et al. Sprint Resisted and Assisted Priming for Peak Performance. J Strength Cond Res. 2023; doi: 10.1519/JSC.0000000000004557. 37639672

[41] Holmberg PM, Harrison PW, Jenkins DG, et al. Factors Modulating the Priming Response to Resistance and Stretch--Shortening Cycle Exercise Stimuli. Strength Cond J. 2023; 45:188–206. doi: 10.1519/SSC.0000000000000728.

[42] Nutt F, Hills SP, Russell M, et al. Morning resistance exercise and cricket-specific repeated sprinting each improve indices of afternoon physical and cognitive performance in professional male cricketers. J Sci Med Sport. 2021; doi: 10.1016/j.jsams.2021.08.017. 34535402

[43] Zabaloy S, Freitas TT, Pareja-Blanco F, et al. Narrative Review on the Use of Sled Training to Improve Sprint Performance in Team Sport Athletes. Strength Cond J. 2023; 45.

[44] Morin JB, Petrakos G, Jiménez-Reyes P, et al. Very-Heavy Sled Training for Improving Horizontal-Force Output in Soccer Players. Int J Sports Physiol Perform. 2017; 12:840–844. doi: 10.1123/IJSPP.2016-0444. 27834560

[45] Morin JB, Bourdin M, Edouard P, et al. Mechanical determinants of 100-m sprint running performance. Eur J Appl Physiol. 2012; 112:3921–3930. doi: 10.1007/s00421-012-2379-8. 22422028

[46] Suchomel TJ, Sato K, Deweese BH, et al. Potentiation Effects of Half-Squats Performed in a Ballistic or Nonballistic Manner. J Strength Cond Res. 2016; 30:1652–1660. doi: 10.1519/JSC.0000000000001251. 26544089

